# Whole-Brain Monosynaptic Afferent Inputs to Basal Forebrain Cholinergic System

**DOI:** 10.3389/fnana.2016.00098

**Published:** 2016-10-10

**Authors:** Rongfeng Hu, Sen Jin, Xiaobin He, Fuqiang Xu, Ji Hu

**Affiliations:** ^1^Center for Neuron and Disease, Frontier Institute of Science and Technology, Xi’an Jiaotong UniversityXi’an, China; ^2^Center for Excellence in Brain Science, Wuhan Institute of Physics and Mathematics, Chinese Academy of SciencesWuhan, China; ^3^School of Life Science and Technology, ShanghaiTech UniversityShanghai, China

**Keywords:** basal forebrain cholinergic system, whole-brain mapping, monosynaptic inputs, rabies viruses, learning and memory

## Abstract

The basal forebrain cholinergic system (BFCS) robustly modulates many important behaviors, such as arousal, attention, learning and memory, through heavy projections to cortex and hippocampus. However, the presynaptic partners governing BFCS activity still remain poorly understood. Here, we utilized a recently developed rabies virus-based cell-type-specific retrograde tracing system to map the whole-brain afferent inputs of the BFCS. We found that the BFCS receives inputs from multiple cortical areas, such as orbital frontal cortex, motor cortex, and insular cortex, and that the BFCS also receives dense inputs from several subcortical nuclei related to motivation and stress, including lateral septum, central amygdala, paraventricular nucleus of hypothalamus, dorsal raphe, and parabrachial nucleus. Interestingly, we found that the BFCS receives inputs from the olfactory areas and the entorhinal–hippocampal system. These results greatly expand our knowledge about the connectivity of the mouse BFCS and provided important preliminary indications for future exploration of circuit function.

## Introduction

Acetylcholine is very important for brain function and has the ability to reconfigure the brain network drastically (Bargmann, 2012). The basal forebrain cholinergic system (BFCS) has been related to brain states and believed to regulate several important behaviors, remarkably involving sleep, attention, decision making (Kilgard, 2003; Yeomans, 2012; Chubykin et al., 2013; Irmak and De Lecea, 2014; Shi et al., 2015). The dysfunction of BFCS is related to neurological disorders such as Alzheimer’s disease (AD) and schizophrenia (Cuello et al., 2010; Bohnen and Albin, 2011; Marra et al., 2012; Banuelos et al., 2013; Burke et al., 2013). Overall, BF nuclei show heterogeneous (Zaborszky et al., 2012). Different type of BF neurons have variable physiological properties and response dynamics to stimuli, representing their specific repertoire of afferent inputs (Hangya et al., 2015). The BFCS makes up a minority of the cell population in the BF (Duque et al., 2007; Wu et al., 2014), in addition to the glutamatergic, GABAergic, and peptidergic neurons (Woolf et al., 1986; Zaborszky et al., 1986, 2012; Depboylu et al., 2012). Traditional tracing approaches have been important in the classification of the major afferents inputs to the BF (Zaborszky et al., 1991). Nevertheless, a full understanding of whole-brain inputs to the BFCS has been hampered because of the inherent limitations of conventional tracing methods. For example: (1) they cannot distinguish the cell types, such as cholinergic or non-cholinergic cells in the BF; (2) passage fibers through or near target structures may absorb the tracers and this may cause non-specificity; and (3) the efficiency of traditional tracers are relatively low. Therefore, in order to overcome these limitations and map the whole-brain afferent inputs to the BFCS, it is necessary to adopt the modern viral tracing system.

Recently, the genetically modified rabies virus has been used to map the monosynaptic inputs to a genetically defined neuronal subtype (Wickersham et al., 2007). It could be applied to characterize the whole-brain presynaptic parterres of a specific type of neurons within a complicated neural network (Wickersham et al., 2007; Wall et al., 2013; Ogawa et al., 2014; Pollak Dorocic et al., 2014; Weissbourd et al., 2014; Grealish et al., 2015). Here, we utilized such viral tracing system to map the whole-brain afferent inputs of the BFCS and discovered that it directly integrates information from several important nuclei. We identified the direct synaptic inputs from neocortex and olfactory areas in the regulation of cholinergic neurons. Also, we discovered that entorhinal–hippocampal system directly project to the BFCS. In addition, we described the presence of direct afferent inputs from several subcortical circuits which are important in regulation of motivation and stress, including lateral septum (LS), central amygdala (CeA), paraventricular nucleus of hypothalamus (PVH), DRN, and PBN. Our findings should be valuable to guide the further investigation of the functional role of the BFCS underlying the normal and neurological disease conditions.

## Materials and Methods

### Animals

All procedures were approved by Institutional Animal Care and Use Committees. Experiments were performed exactly as approved by the IACUC at ShanghaiTech University and Center for Excellence in Brain Science, Wuhan Institute of Physics and Mathematics, the Chinese Academy of Sciences, China. The BAC-transgenic ChAT-Cre mice, obtained from MMRRC (Davis, CA, USA), was utilized throughout the study. These mice were back crossed with the C57BL/6J mice. And the obtained mice were used in the study. The mice were raised under 12/12 day/night cycle at the temperature of 22–25°C, with *ad libitum* access to rodent chow and water freely available in environmentally controlled conditions. Four adult mice of either sex (2–3 months old) were used in the study, and we also used the wild-type C57BL/6J mice wild-type C57BL/6J mice as control.

### Viruses and Surgery

AAV-CAG-DIO-TVA-GFP (AAV2/9, 1.7 × 10^13^ genomic copies per ml), AAV-CAG-DIO-RG (AAV2/9, 6.8 × 10^12^ genomic copies per ml), and EnvA-pseudotyped, glycoprotein (RG)-deleted and DsRed-expressing rabies virus (RV-EvnA-DsRed, RV; 5.0 × 10^8^ genomic copies per ml) were packaged and provided by F. Xu’ Lab (Wuhan, China). Briefly, the initial rabies viruses SAD-1G- mCherry (EnvA) and the rabies propagation and titering cell lines were both provided by E.M. Callaway at Salt Institute. The detailed production and concentration procedures for modified rabies viruses were previously described (Osakada et al., 2011). Cre-dependent helper virus vectors, AAV-CAG-DIO-TVA-GFP, and AAV-CAG-DIO-RG were also produced for retrograde transsynaptic tracing (Wang et al., 2015). The CAG promoter of these two plasmids were sub-cloned from AAV-CAG-GFP-ires-CRE plasmid (Addgene plasmid 48201). And the coding region of TVA-GFP and RG were obtained from the AAV-EF1a-FLEX-GT plasmid (Addgene plasmid 26198) and were separately constructed into the DIO cassette of the plasmid pAAV-EF1a-DIO-hChR2 (H134R)-EYFP (Addgene plasmid 20298).

Surgery procedures were generally followed by previous studies (Liu et al., 2014; Hu et al., 2016). Briefly, mice were anesthetized by intraperitoneal injection of atropine (0.05 mg/kg) and pentobarbital (80 mg/kg), kept warm (37°C) with an electric heating pad (BrainKing Biotech), and mounted in a stereotaxic holder in order to adjust the skulls of experimental mice parallel with the reference panel. Using a microsyringe pump (Nanoliter, 2000 Injector, WPI), 50–100 nl of AAV-CAG-DIO-TVA-GFP and AAV-CAG-DIO-RG were stereotaxically injected (46 nl min^-1^) into the unilateral BF (AP: +0.1 mm, ML: +1.25 mm, DV: 5.3 mm), followed by an additional 5 min to allow diffusion of viral particles away from the injection site before slowly withdrawn. Two weeks later, 300 nl of EnvA-pseudotyped, glycoprotein (RG)-deleted and DsRed-expressing rabies virus (RV-EvnA-DsRed, RV; 5.0 × 10^8^ genomic copies per ml) was injected into the same area.

### Histology and Image Analysis

One week after injection of rabies virus, mice were perfused with PBS followed by 4% paraformaldehyde (PFA) in PBS. After 24 h of post-fixation in 4% PFA, coronal brain slices at 60 μm thickness were prepared using a cryostat (Leica CM1900). Every third section was counterstained with DAPI (Molecular Probes, Eugene, OR, USA) and visualized with an Olympus VS120 microscope. Further data analyses were performed using Olympus analysis software and ImageJ software. For quantifications of subregions, boundaries were based on the Allen Institute’s reference atlas (Lein et al., 2007). We selectively analyzed the retrogradely labeled dense areas. The proportion of total inputs was shown in (**Figure 3**) and the input densities were expressed as cells/mm^2^ (**Figure 3**).

To characterize the inputs from the orbitofrontal cortex and PC, we immunostained the brain slices containing the two areas with the antibody anti-parvalbumin (PV) according to the previous study (Hu et al., 2016). Briefly, the sections were first blocked with 3% BSA in PBS-0.3% Triton X-100 for 30 min and incubated with the primary rabbit anti-PV antibody (1:200, Abcam) for 48 h at 4°C. After washing, the sections were incubated with second antibody (Alexa Fluor 488 goat anti-rabbit, Abcam; 1:1000, 2 h) at room temperature. Imaging and analysis of images were carried out with a digital slide scanner (CarlZeiss, LSM710).

## Results

### Identification of Monosynaptic Inputs onto the BF Cholinergic Neurons Using a Rabies-Based System

**FIGURE 1:**
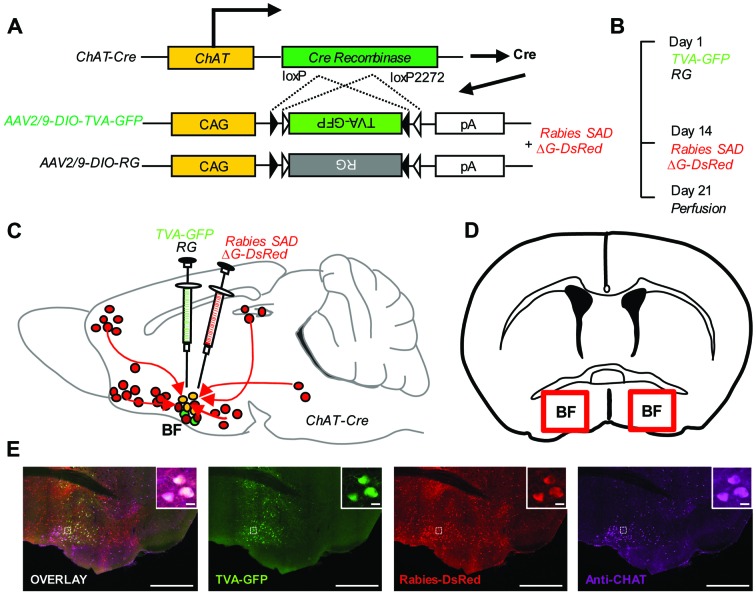
**Monosynaptic inputs to the BFCS using rabies-based transsynaptic tracing approach. (A)** AAV helper viruses with Cre-dependent expression of TVA receptor and RG. Genetically modified rabies virus is pseudotyped with EnvA. The RG gene is replaced by DsRed. **(B)** Experimental timeline. **(C,D)** Sagittal section **(C)** and coronal section **(D)** for schematic illustration of AAV helper virus (green) and rabies virus (red) injection into the BF of ChAT-Cre mice. **(E)** Example of injection site: scale bar, 2 mm. Zoom-in view of injection site: scale bar, 10 μm.

We genetically targeted the BFCS based on a transgenic mouse line expressing Cre recombinase in cholinergic neurons (ChAT-Cre mice) (Gong et al., 2007). In ChAT-Cre mice, we applied a genetically engineered viral system to map the whole-brain afferent inputs to the BFCS. We first co-expressed the avian receptor TVA and the rabies glycoprotein G (RG) in the BFCS neurons, which is achieved through injection of two AAV-DIO helper viruses (AAV-DIO-TVA-GFP and AAV-DIO-RG; **Figure 1A**) into the BF (**Figures 1C,D**) of ChAT-Cre mice (Cardin et al., 2009). Two weeks later, we injected the genetically modified rabies virus with the avian virus envelope protein (EnvA) (**Figures 1A–C**). In addition, the endogenous RG of this rabies virus has been genetically substituted to express the fluorescent protein DsRed (**Figure 1A**). So this rabies virus can only infect cells expressing the TVA receptor, which should be restricted to Cre-expressing BFCS neurons (**Figure 1E**, Supplementary Figure S1). TVA-GFP signals were colocalized with CHAT immunoreactivity in the majority (~90%) of the neurons in the BF (**Figure 1E**, Supplementary Figure S1), confirming the accuracy of CHAT driver mouse lines. Taken together, we could use this modern technique to map the whole-brain monosynaptic afferent inputs to the BFCS (**Figure 1E**).

### Overview of the Whole-Brain Inputs to BFCS Neurons

**FIGURE 2:**
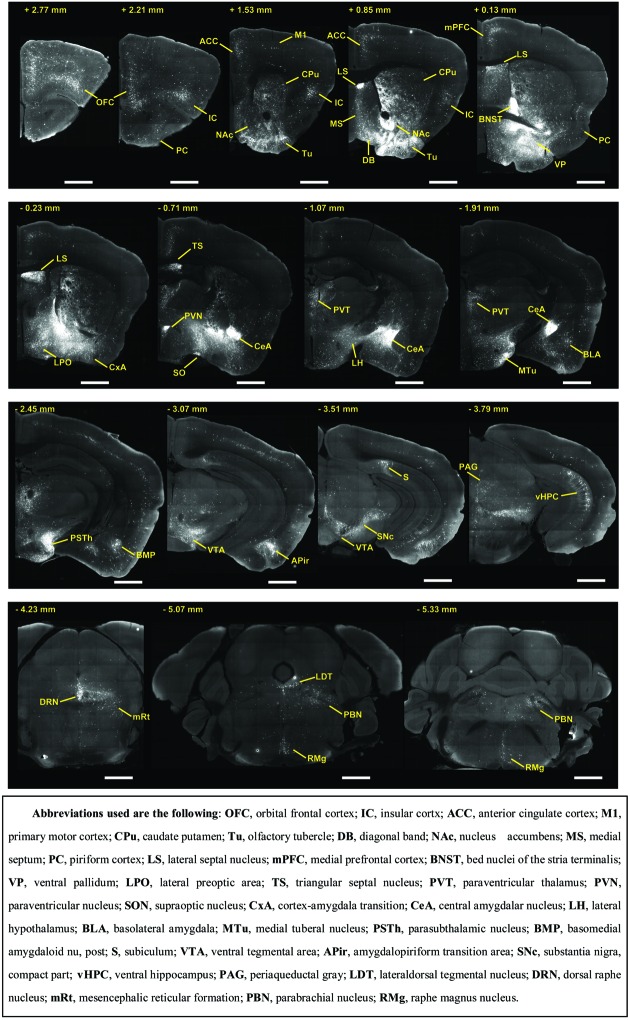
**Representative coronal sections showing labeling of monosynaptic inputs to the BFCS.** Scale bar: 1 mm.

**FIGURE 3:**
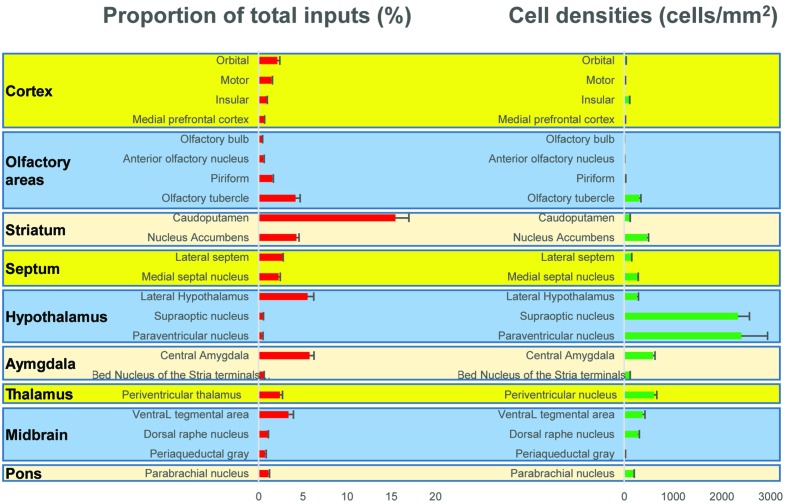
**Statistical analysis of the monosynaptic inputs (Left) Proportion of input neurons for BF cholinergic neurons.** The values are the normalized ratio of the cell number in each area against the total number of input neurons. Error bars represent the SEM. *n* = 4 ChAT-Cre mice. **(Right)** Cell density of input neurons in each brain area.

To outline the whole-brain monosynaptic afferent inputs to the BFCS, we explored serial coronal brain sections (**Figure 2**, Supplementary Figure S2) after viral tracing. Sections from typical ChAT-cre (**Figure 2**, Supplementary Figure S2) brains showed that DsRed^+^ afferent input neurons are largely located in ipsilateral forebrain and midbrain nuclei. We found that the BFCS neurons integrate inputs from a wider than previously believed brain regions. For example, the BFCS received direct synaptic inputs from neocortex, olfactory areas, entorhinal–hippocampal system directly project to the BFCS. Also, we found that many motivation and stress related subcortical nuclei provided direct input to the BFCS, such as the LS, CeA, PVH, DRN, and PBN. Finally, many regions of the brain were sparsely labeled, including thalamus and some hindbrain nuclei. To describe the whole-brain distribution of the afferent inputs of the BFCS, we allocated each brain into 22 areas of interest and then counted the input numbers and calculated the input densities (**Figure 3**). We also carried out the immunostaining experiments to characterize the inputs from the orbitofrontal cortex (OFC) and PC, and the results showed that the neurons in the OFC and PC that target the BFCS are not parvalbumin-positive neurons (Supplementary Figure S3).

### BFCS Neurons Receive Widespread Cortical Inputs

**FIGURE 4:**
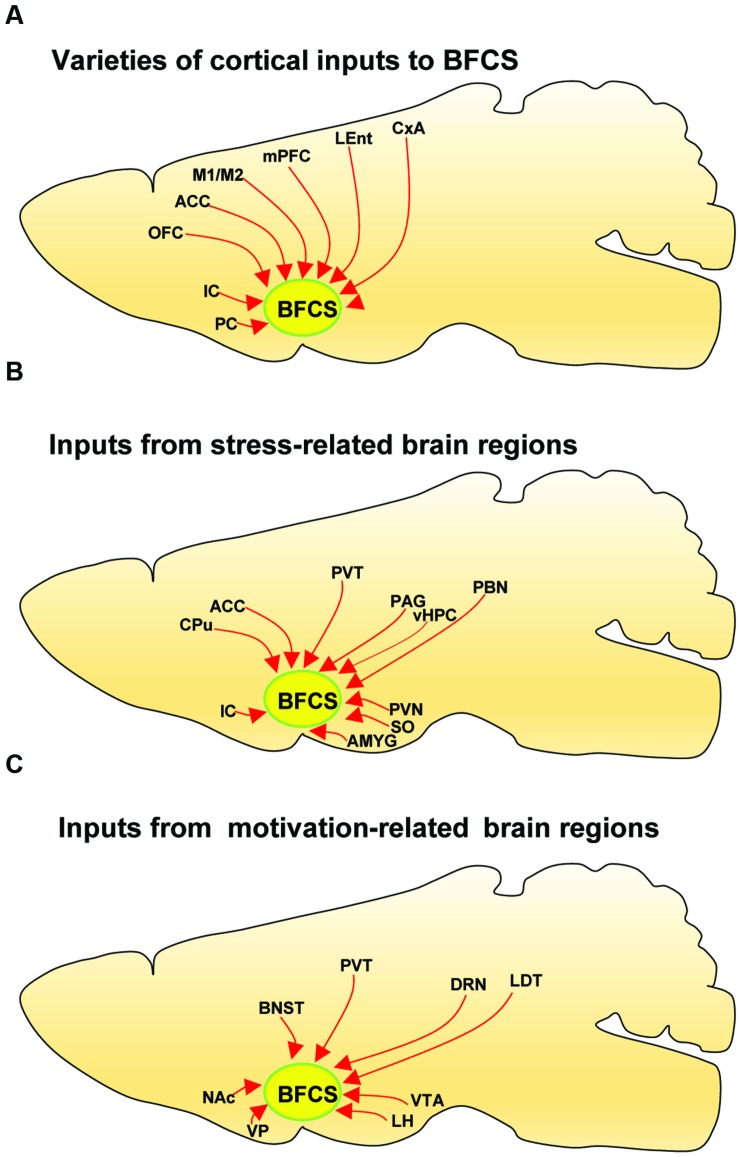
**Summarized connectivity of the BFCS. (A)** Schematic summary of cortical inputs to the BFCS. **(B)** Schematic summary of inputs from stress-related brain regions to the BFCS. **(C)** Schematic summary of inputs from motivation-related brain regions to the BFCS.

The BFCS has been suggested to control cortical states through projections across the whole neocortical areas (Sarter and Bruno, 2000; Pinto et al., 2013; Kimura et al., 2014; Ljubojevic et al., 2014). Also, there was evidence demonstrating that the BF received input back from the cortex (Zaborszky et al., 1997). However, the detailed organizations of cortical areas which monosynaptically target BF neurons was uncharacterized, especially in a cell-type-specific manner. Here, we found that across the neocortical areas, the most abundant labeling was found in the ipsilateral anterior cortex, including OFC, insular cortex (IC), primary motor cortex (M1), mPFC, and frontal association (**Figures 2** and **4A**), demonstrating that BFCS is one of the direct targets of the anterior cortexes (**Figure 3**). In contrast, sparse labeling was found in the posterior neocortical areas. To our surprise, nearly all posterior neocortical areas project to the BFCS, which most of the retrograde labeled neurons are located at layer 5 (**Figure 2**, Supplementary Figure S2). The bidirectional connectivity between the cortexes and the BF has attracted great interest as a circuit involved in modulating decision making, cortical arousal, and learning and memory (Ramanathan et al., 2009; Martinowich et al., 2012; Paolone et al., 2012; Bloem et al., 2014). Our results should provide the foundations for further investigation of circuit mechanism of those important behaviors.

### BFCS Neurons Receive Widespread Olfactory Inputs

Olfactory cues are essential for animal survival (Ligout et al., 2004; Asahina et al., 2008). Here, we found that the BFCS received significant monosynaptic inputs from olfactory areas, including olfactory bulb (OB), anterior olfactory nucleus (AON) and PC (**Figures 2** and **3**). Among them, the PC is the largest inputs to the BFCS neurons. The PC is divided into anterior (aPC) and posterior (pPC) portions, and the former receives more afferent input from the OB and fewer associational inputs, and links more strongly to the outside world (Bekkers and Suzuki, 2010). Our results revealed that both aPC and pPC both project to the BFCS neurons. The OB sends fewer projections to the BFCS neurons, mainly from the Mitral/Tufted cell layer. The AON plays a pivotal but relatively poorly understood role in the processing of odor information. We observed that many AON neurons sent direct inputs onto the BFCS neurons, which may provide the new perspectives for the functional role of AON.

### BFCS Neurons Receive Inputs from Entorhinal–Hippocampal System

The entorhinal–hippocampal system, which consists of the hippocampus, perirhinal cortex, the dentate gyrus, the subicular areas and the entorhinal cortex (EC), is one of the most important brain network for memory and emotion (Buzsaki and Moser, 2013). It plays an important role in declarative memories and in particular spatial memories including memory formation, memory consolidation, and memory optimization in sleep (Buzsaki and Moser, 2013). Here, we found that the EC and vHPC sent strong projections to the BFCS neurons. Notably, the BFCS neurons received dense inputs from the dorsal subiculum (**Figure 2**, Supplementary Figure S2). These results suggested that there was crosstalk between cholinergic system and hippocampal system, which should be considered in the future investigation of the role of acetylcholine in learning and memory and emotion.

### Multiple Stress-Related Brain Areas Target BFCS

Stress has been implicated in the emergence of numerous pathologies, including depression and anxiety disorders, and as a behavioral modifier that affects fundamental processes, such as learning and memory (Bains et al., 2015; Hollon et al., 2015). Interestingly, our results demonstrated that the BFCS receives strong inputs from many stress-related brain areas (**Figures 2**, **3**, and **4B**). One of the prominent retrograde labeled brain region is the amygdala. The amygdala has long been associated with emotion (Janak and Tye, 2015). Understanding the intricacies of amygdala circuitry is of tremendous importance given that the amygdala is implicated in a wide range of disease states, including addiction, autism and anxiety disorders (Janak and Tye, 2015). Conventional retrograde tracing studies have determined that projections from amygdala to BF originate primarily from the CeA, with limited inputs from the other amygdala nuclei. Both the central and basolateral nuclei of the amygdala are known to innervate BF, but the specificity and relevance of this input stream is not well understood. In accordance with previous studies, we detected the majority of DsRed-labeled amygdala input neurons in the CeA (**Figures 2** and **3**), and we just found few DsRed-labeled neurons in basolateral amygdala (BLA) and medial amygdala nucleus (MeA) (**Figures 2** and **3**, Supplementary Figure S2).

In addition to amygdala, there are several other stress-related brain regions sending strong projections to the BFCS, such as LS, PVT, vHPC, PVH, SON, periaqueductal grey matter (PAG), and PBN. These structures are involved in the autonomic, hormonal and behavioral responses to stress and fear. Our results may provide insights for the further investigation of circuit mechanism of depression and anxiety disorder.

### Motivation-Related Brain Regions Send Projections to BFCS

The VP is a crucial node in the ventral striatopallidal circuits underlying reward-related behaviors, with major reciprocal connections to the NAc (Mahler et al., 2014). Our monosynaptic tracing data showed that the VP and NAc both strongly target the BFCS (**Figures 2** and **4C**). The bed nucleus of the stria terminalis (BNST), a neural component of the extended amygdala (Jennings et al., 2013), is a key integrator of diverse motivational states through its interactions with various synaptic targets, including the LH, a brain region historically implicated in reward and motivation (Harris et al., 2005). Interestingly, our results demonstrated that these two brain areas densely targeted the BFCS (**Figures 2** and **4C**), indicating that BFCS may be regulated by motivational events. Also, the limbic system, including limbic amygdala, conveys affective value information to the BFCS (**Figure 2**). Both traditional tracing techniques and rabies virus-based monosynaptic tracing approaches have discovered that BF neurons project their axons into the brain regions controlling the reward system, including VTA, DRN, and LDT (Zaborszky et al., 2012). Here, our results showed that these brain areas also send dense projections to the BFCS (**Figures 2** and **4C**), indicating that the BFCS bi-directionally connects multiple motivation-related brain regions to modulate the behaviors.

## Discussion

The BFCS is essential to some of the most vital functions of the brain, including attention, arousal, decision making, and learning and memory. To understand the circuit mechanism of how the BFCS modulate this broad spectrum of behavior, it is crucial to explore the afferent inputs impacting the activity of the BFCS (Alheid, 2003). Traditional mapping of inputs to the BF was based on conventional retrograde tracing techniques, which does not allow for identification of specific afferent of cholinergic neurons (Wall et al., 2013; Ogawa et al., 2014; Pollak Dorocic et al., 2014; Weissbourd et al., 2014). In the present study, our rabies-based system efficiently mapped the extensive whole-brain afferent inputs of the BFCS, especially inputs from a wide range of cortical regions, olfactory areas, entorhinal–hippocampal system, stress-related brain regions, and motivation-related brain nuclei (**Figures 2–4**). Comparing to previous results using traditional methods, our findings are more precise and efficiency, which should provide a comprehensive map of the presynaptic partners that control the BFCS.

### Implications for the Role of BFCS in Attention and Decision Making

Basal forebrain cholinergic system has long been implicated in attention (Dalley et al., 2004; Martinez and Sarter, 2004; Sarter et al., 2005, 2014; Sarter, 2007; Broussard et al., 2009; Fadel and Burk, 2010; Paolone et al., 2012; D’Souza and Vijayaraghavan, 2014; Ljubojevic et al., 2014; Oros et al., 2014; Parikh et al., 2014), an important behavioral and cognitive process through selectively concentrating on a discrete aspect of information (Paolone et al., 2012). Previous studies showed that animals with restricted BFCS lesion causes disruption in attentional processing (Muir et al., 1992a,b, 1993, 1994; McGaughy and Sarter, 1999; Waite et al., 1999; Dalley et al., 2004). The higher-order association cortices in the temporal and parietal lobes and prefrontal cortex (PFC) also have been found to mediate aspects of attention (Craft et al., 2005). In addition, previous findings showed PFC areas support appropriate decision-making (Kennerley and Walton, 2011; Steiner and Redish, 2014; Siegel et al., 2015). Our result revealed that BFCS is one of the major target of PFC. These two areas are interconnected to support attentional processing. Cortical cholinergic neurotransmission is regulated on multiple timescales to mediate the detection of behaviorally significant cues and to support cognitive performance (Parikh et al., 2007).

### Implications for the Role of BFCS in Sleep and Arousal

Arousal is considered to be play an important role in the pathophysiology of sleep disorders. The BFCS is recognized as important site of sleep-wake regulation (Koyama, 2012; Kalinchuk et al., 2015). The BFCS heavily projects to the entire cortex and limbic system, excites the target cells and involves in the regulation of waking and REM sleep (Jones, 1993; Rodrigo-Angulo et al., 2008; Platt and Riedel, 2011). Optogenetic activation of BFCS induce sleep-awake transition (Han et al., 2014). Here, we showed that BFCS receive widespread input from hypothalamic nuclei, including the preoptic areas and LH, and both have implicated in sleep and arousal regulations (Szymusiak et al., 2007). Our result may provide explanations for how BFCS participate the neural network for brain state and sleep regulation.

### Implications for the Role of BFCS in Reward System

Lesion and stimulation of the VTA increases cholinergic activity in the rat brain (Majkutewicz et al., 2010). Both traditional tracing technique and rabies virus-based monosynaptic tracing approach have discovered that BF neurons project their axons into the brain regions controlling the reward system, including VTA, DRN, NAc, and OFC (Zaborszky et al., 2012). Here, we found that these brain areas also send projections to the BFCS. These collective data indicate that the BF interconnects with the brain reward system, implying that the BFCS is an important node in this system. Also, increasing evidence indicated that the VP, a subregion of the BF, plays vital roles in reward and motivation [see reviewed in Smith et al. (2009)].

Collectively, our viral tracing results provide new perceptive for the future exploration of circuit mechanism underlying the function of the BFCS, such as: (1) The function of olfactory input (directly from OB, AON, Tu, and PC) to the BFCS; (2) The function of feedback cortical projections onto the BFCS. The BFCS plays important role in cortical activation, it should be interesting to examine how cortical neurons (particularly, layer 5 neurons) impact the BFCS; (3) The physiological and behavioral function of entorhinal–hippocampus projections to the BFCS; (4) The comprehensive functions of the BFCS in motivation and reward; (5) Considering the dense inputs from stress-related regions, it should be important to examine the role of BFCS in stress and emotion regulation. Therefore, further functional and behavioral evidence is of great need and interest to be provided.

## Author Contributions

JH and RH conceptualized the project. RH performed the majority of experiments. SJ, XH, and FX provided the viruses used in this study. JH and RH analyzed the data and wrote the manuscript with the participation of all other authors.

## Conflict of Interest Statement

The authors declare that the research was conducted in the absence of any commercial or financial relationships that could be construed as a potential conflict of interest.

## Abbreviations

ACC: anterior cingulate cortex
AMYG: amygdala
BNST: bed nuclei of the stria terminalis
CPu: caudate putamen
CxA: cortex-amygdala transition
DRN: dorsal raphe nucleus
IC: insular cortx
LDT: lateraldorsal tegmental nucleus
LEnt: lateral entorhinal cortex
LH: lateral hypothalamus
M1/2: motor cortex
mPFC: medial prefrontal cortex
NAc: nucleus accumbens
OFC: orbital frontal cortex
PAG: periaqueductal gray
PBN: parabrachial nucleus
PC: piriform cortex
PVN: paraventricular nucleus of the hypothalamus
PVT: paraventricular thalamus
SON: supraoptic nucleus
vHPC: ventral hippocampus
VP: ventral pallidum
VTA: ventral tegmental area
