# Supplementary figures and images for: Whole-Brain Monosynaptic Afferent Inputs to Basal Forebrain Cholinergic System
Source: Front Neuroanat. 2016 Oct 10;10:98. doi: 10.3389/fnana.2016.00098 (PMC5056182; doi:10.3389/fnana.2016.00098)

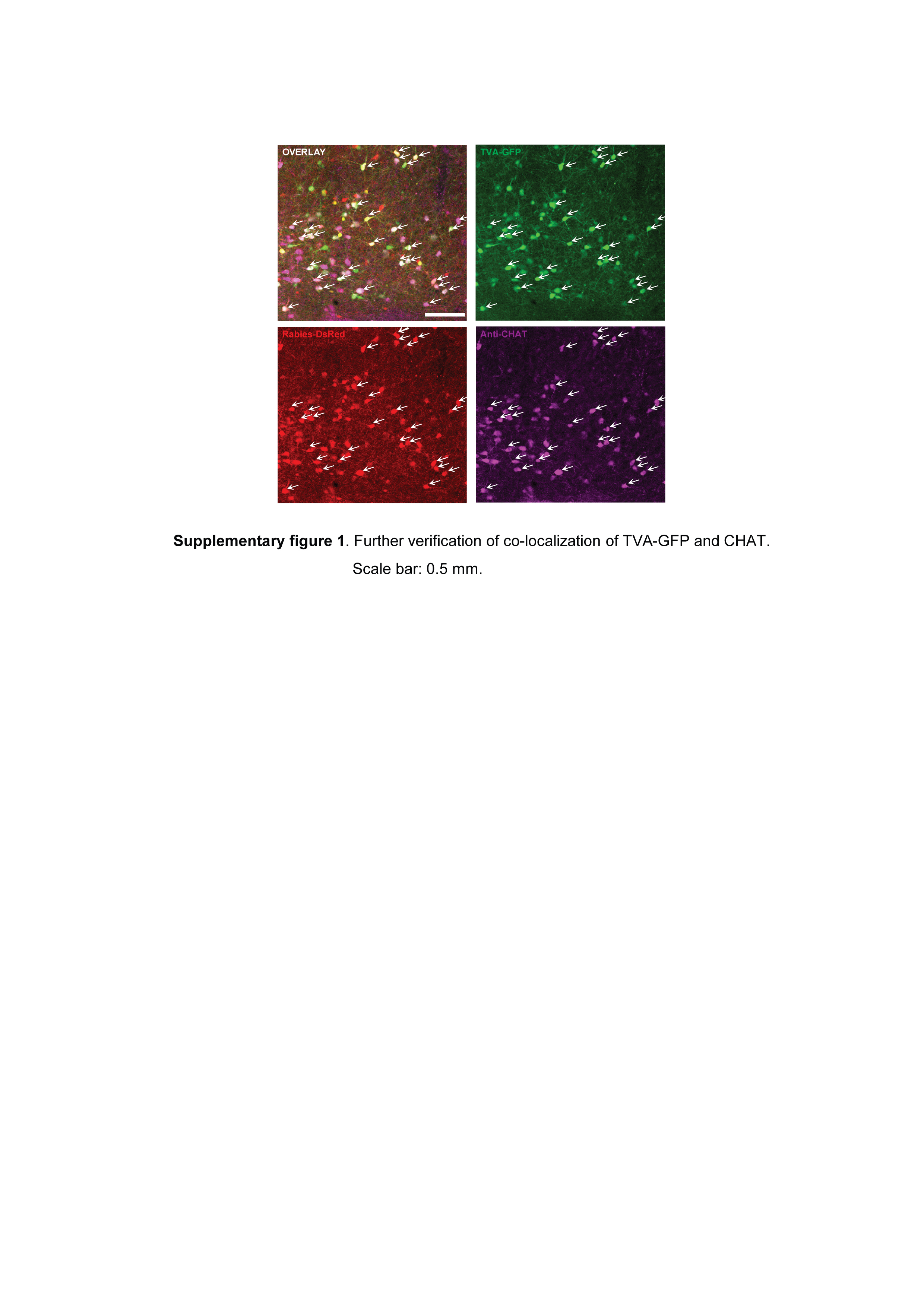

Supplement: Supplementary file 1 [file Image_1.JPEG]

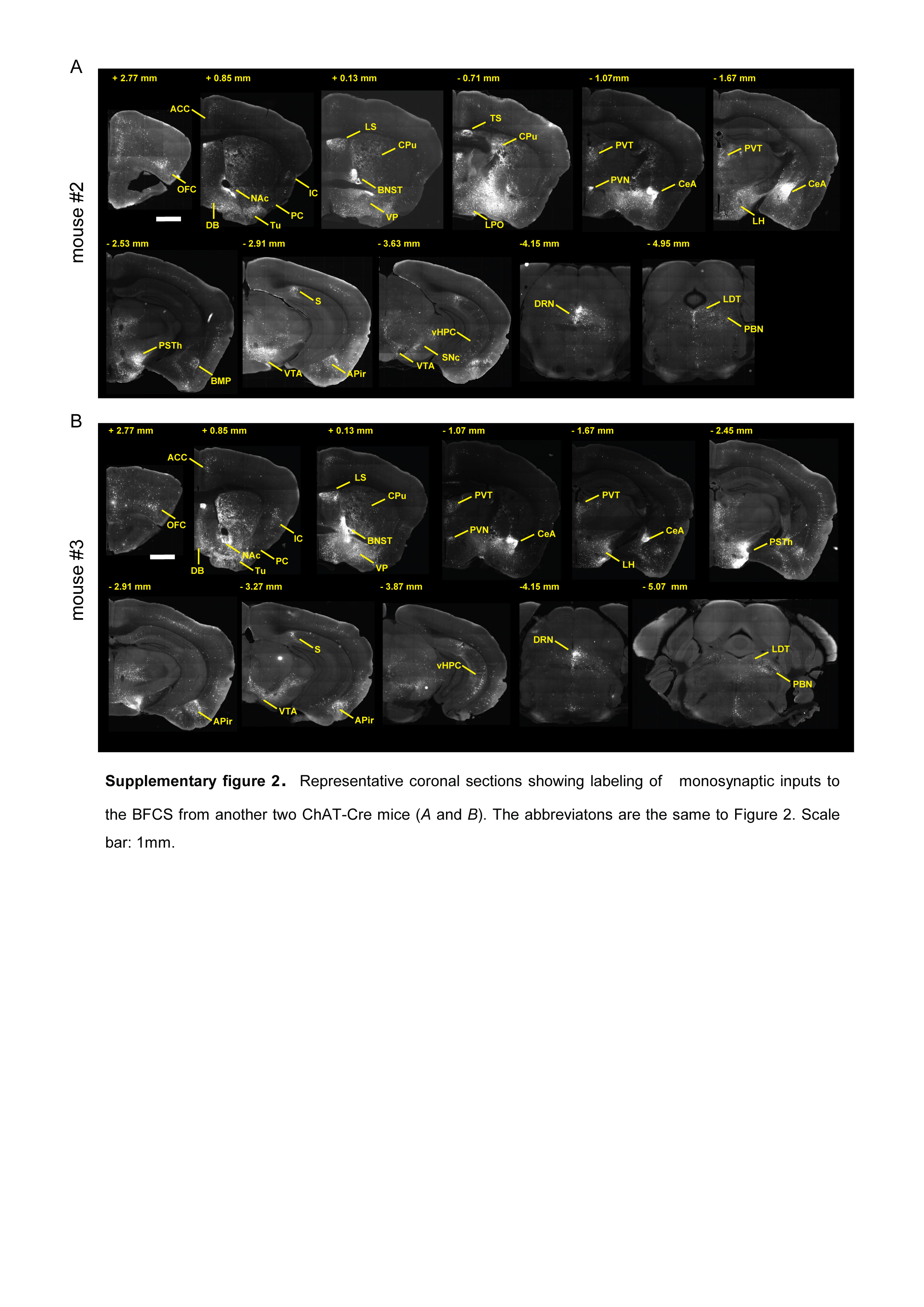

Supplement: Supplementary file 2 [file Image_2.JPEG]

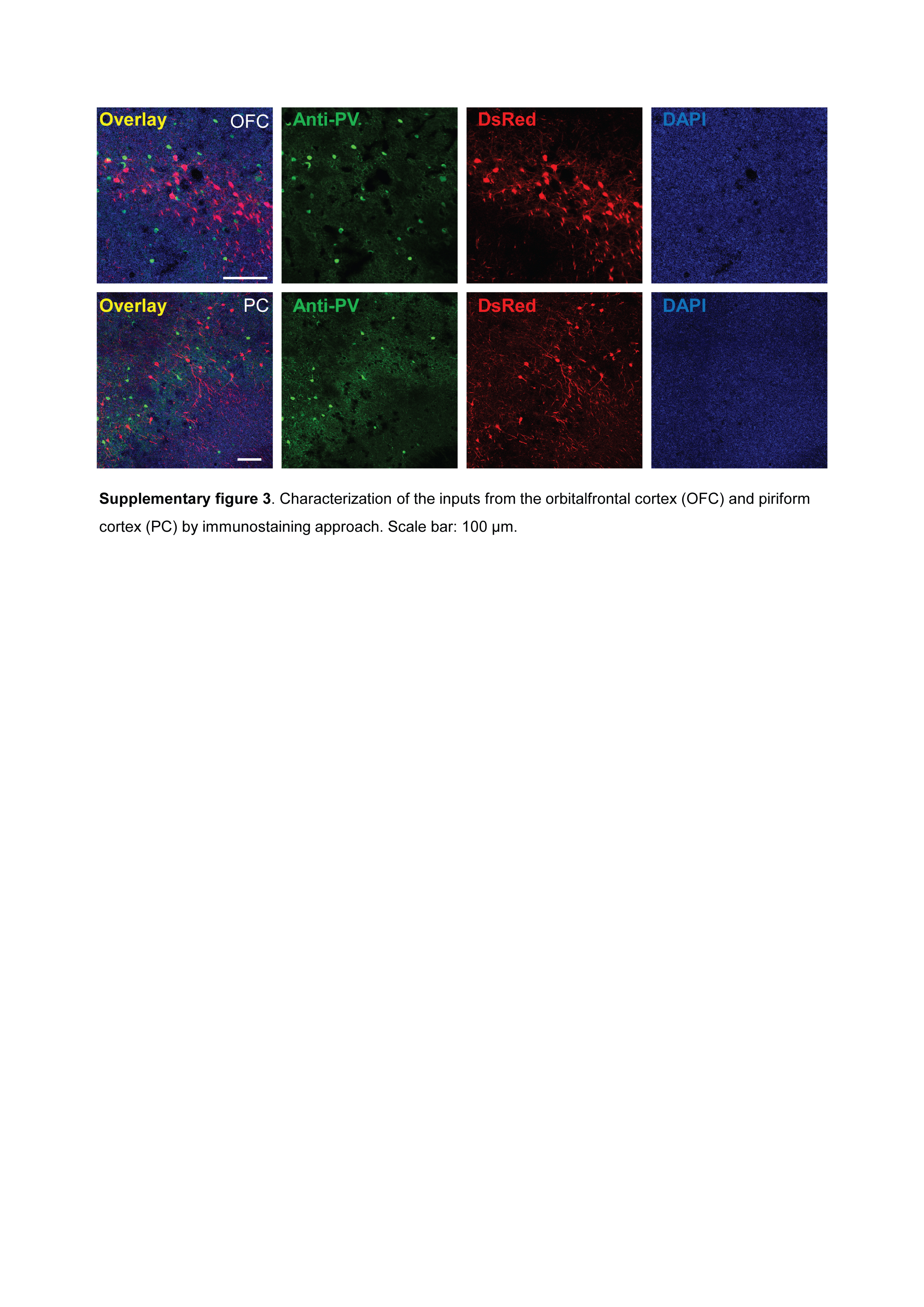

Supplement: Supplementary file 3 [file Image_3.JPEG]
